# Evolutionary Analysis of JAZ Proteins in Plants: An Approach in Search of the Ancestral Sequence

**DOI:** 10.3390/ijms20205060

**Published:** 2019-10-12

**Authors:** Adrián Garrido-Bigotes, Felipe Valenzuela-Riffo, Carlos R. Figueroa

**Affiliations:** 1 Laboratorio de Epigenética Vegetal, Facultad de Ciencias Forestales, Universidad de Concepción; Concepción 4070386, Chile; adrigarrido@udec.cl; 2 Institute of Biological Sciences, Campus Talca, Universidad de Talca, Talca 34655488, Chile; felvalenzuela@utalca.cl

**Keywords:** Jasmonate-ZIM domain, JAZ repressors, Jas domain, TIFY, degron, phylogenetic analysis, ancestral sequences

## Abstract

Jasmonates are phytohormones that regulate development, metabolism and immunity. Signal transduction is critical to activate jasmonate responses, but the evolution of some key regulators such as jasmonate-ZIM domain (JAZ) repressors is not clear. Here, we identified 1065 JAZ sequence proteins in 66 lower and higher plants and analyzed their evolution by bioinformatics methods. We found that the TIFY and Jas domains are highly conserved along the evolutionary scale. Furthermore, the canonical degron sequence LPIAR(R/K) of the Jas domain is conserved in lower and higher plants. It is noteworthy that degron sequences showed a large number of alternatives from gymnosperms to dicots. In addition, ethylene-responsive element binding factor-associated amphiphilic repression (EAR) motifs are displayed in all plant lineages from liverworts to angiosperms. However, the cryptic MYC2-interacting domain (CMID) domain appeared in angiosperms for the first time. The phylogenetic analysis performed using the Maximum Likelihood method indicated that JAZ ortholog proteins are grouped according to their similarity and plant lineage. Moreover, ancestral JAZ sequences were constructed by PhyloBot software and showed specific changes in the TIFY and Jas domains during evolution from liverworts to dicots. Finally, we propose a model for the evolution of the ancestral sequences of the main eight JAZ protein subgroups. These findings contribute to the understanding of the JAZ family origin and expansion in land plants.

## 1. Introduction

Jasmonates (JAs) are phytohormones that regulate the defense responses, growth and development, fertility and reproduction, as well as the biosynthesis of secondary metabolites in terrestrial plants [1]. Their biosynthesis and canonical signaling pathways have been well characterized in vascular plants [2]. Specifically, the JA-signaling pathway is critical for suitable responses to development or environmental stress. The activation of the JA-signaling pathway starts with the perception of the bioactive JA, jasmonoyl-isoleucine (JA-Ile), which is a necessary step for the activation of JA responses. The perception mechanism is mediated by the protein co-receptor complex CORONATINE INSENSITIVE1 (COI1)–jasmonate ZIM-domain (JAZ) in model plants of vascular plants groups such as *Arabidopsis thaliana* and *Fragaria vesca* [3,4], among others. The presence of JA-Ile leads to JAZ protein degradation by the proteasome, and the release of MYC transcription factors (TFs), which are master regulators of JA responses [5,6,7,8]. Besides, additional proteins of the JA-signaling pathway such as the adaptor protein Novel Interactor of JAZ (NINJA), co-repressor proteins, e.g., like TOPLESS (TPLs), histone deacetylases (HDAs), antagonistic TFs, e.g., JASMONATE-ASSOCIATED MYC2-like (JAMs), and MYC2-TARGETED BHLH (MTB) proteins establish a fine-tuning repressor mechanism on MYC TFs [9,10,11,12,13]. In the case of lower plants such as the liverwort *Marchanthia polymorpha*, COI1, JAZ co-repressor and MYC TFs are conserved, although the ligand molecule is dinor-oxophytodienoic acid (dnOPDA) [14,15,16]. Thus, the machinery of perception of the JA-signaling pathway and JAZ repressors seems to be conserved from liverworts to angiosperms, although some structural and functional differences could exist because of the great phylogenetic distance of these lineages.

**Figure 1:**
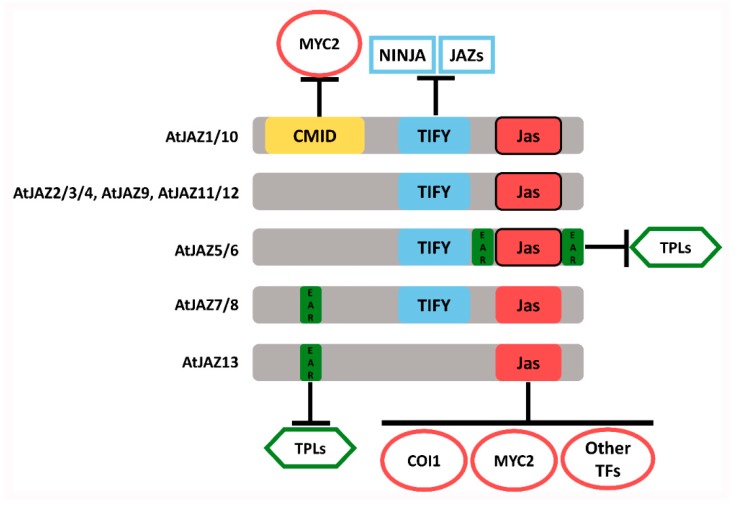
Schematic representation of *Arabidopsis thaliana* JAZ proteins showing the main motifs and domains through which they interact with other proteins (interactions indicated by T-bars and proteins by circles, rectangles and hexagons). Frame boxes of Jas domains indicate the conservation of functional degrons. CMID, Cryptic MYC2-interacting domain; COI1, CORONATINE INSENSITIVE1; EAR, ethylene-responsive element binding factor-associated amphiphilic repression; JAZ, jasmonate ZIM-domain; NINJA, Novel Interactor of JAZ; TFs, transcription factors; TPL, TOPLESS. This figure is based on References [3,9,22,24,25,27,29].

JAZ repressors belonging to the TIFY family have been extensively studied in higher plants [17,18,19,20,21]. In Arabidopsis, this subfamily comprises 13 JAZ proteins (Figure 1) [17,22], while in *M. polymorpha* it only contains a single JAZ protein [14,15]. Normally, these proteins contain the TIFY (ZIM) conserved domain (Figure 1) consisting of 28 amino acid (aa) residues with the highly conserved motif TIF(F/Y)XG in *A. thaliana* [23], which is involved in the JAZ–NINJA and JAZ–JAZ protein interactions (Figure 1) [9,24]. However, some JAZ proteins such as AtJAZ13 lack the TIFY domain (Figure 1) [22]. In addition, all JAZ proteins contain the Jas domain, which is constituted by 26 aa that mediate the COI1–JAZ and MYC2–JAZ interactions (Figure 1) [25]. Generally, the Jas domain contains the degron sequence LPIAR(R/K), which is crucial for the COI1–JA-Ile–JAZ complex formation [3], the Jas motif for interaction with TFs and the nuclear localization signal (NLS) (Figure 1) [25]. Nevertheless, the functionality of JAZ proteins can be different depending on specific protein domains or motifs. For instance, some JAZ proteins own an alternative degron sequence, e.g., JAZ7 and JAZ8 in Arabidopsis, which is not functional, and therefore cannot be degraded by the proteasome [26]. Besides, the additional cryptic MYC2-interacting domain (CMID) has been identified in some JAZ proteins such as *A. thaliana* JAZ1 and JAZ10 proteins [27] (Figure 1), although it is weakly conserved [27]. Finally, ethylene-responsive element binding factor-associated amphiphilic repression (EAR) motifs (LxLxL) are present in some JAZ proteins such as *A. thaliana* JAZ5, JAZ6, JAZ7 or JAZ8 (Figure 1), which are related with the interaction with TPL proteins and the resultant repression [26,28]. Therefore, the diversity of JAZ proteins generated by the absence/presence of these domains or by particular changes in structural and functional domains leads to the regulation of a wide set of responses in higher plants, although a functional redundancy of JAZ repressors has been suggested [29]. In turn, the degron is a key sequence for JA responses, and the canonical degron defined as LPIAR(R/K) has been well studied in *A. thaliana* [3]. In this sequence, the fifth and sixth amino acid residues (from the N- to the C-terminus) are the highest conserved [3,4] and are involved in the interaction with COI1 in the presence of JA-Ile [3]. Moreover, these two residues remain conserved in lower plants such as *M. polymorpha* [14,15], in monocots such as *Oryza sativa* [19], and in angiosperms such as *F. vesca* [18], *Solanum lycopersicum* [17] or *Vitis vinifera* [30]. However, information about degron sequences in other plant lineages is limited.

In general, the evolution of the JA-signaling pathway and JAZ proteins through the plant kingdom has been scarcely explored. However, some evolutionary analysis on the TIFY family and the F-box domain of COI1 receptors’ plant hormone signaling has been performed [21,31]. The signal transduction of JA is not present in algae lineage and appeared in land plants for the first time [31], thus increasing the number and diversity of JAZ proteins. Several authors suggest that the JA-signaling pathway arose from a common ancestor with auxins because the receptors exhibit a high similarity, share TPL proteins such as co-repressors, and the repressors are degraded by the proteasome [2]. Bai et al. [21] reported that the number of proteins that are members of the JAZ family increases as we move along the phylogenetic scale, with lower plants such as bryophytes containing 1–9 members and angiosperms comprising more than 10 members in the JAZ subfamily. On one hand, genome-wide characterization of JAZ proteins has mainly been studied in several families of higher plants, both in monocots such as rice [19] and wheat [20], and in dicots such as cotton [32], strawberry [18], tomato [17] and grape [30], among others. On the other hand, in gymnosperms, as well as in mosses and lycophytes, this protein family has been less studied. Nevertheless, data about the origin and evolution of JAZ proteins through the plant kingdom are still unknown. The large amount of genomic data now available in the plant kingdom allows the study of the evolutionary history of JAZ proteins. The aim of this research was to analyze JAZ proteins’ evolution through the structural characterization of domains and motifs, phylogenetic analysis and reconstruction of the ancestral sequence for JAZ proteins in different lineages of land plants.

## 2. Results

### 2.1. Identification of JAZ Protein Family in the Plant Kingdom

In order to identify JAZ proteins in different lineages of the plant kingdom, *A. thaliana* JAZ proteins were used as queries by using blastp searches against the proteomes included in the PLAZA database. Previously reported JAZ sequences were directly obtained from their respective reports (Table 1). After removing partial sequences, sequences lacking the Jas domain, sequences with additional domains such as PEAPOD (PPD) or GATA zinc-finger (ZML), and redundant sequences, 1065 sequences were identified. In this sense, we identified 183, 6, 196 and 389 new JAZ genes corresponding to gymnosperms, *Amborella trichopoda*, monocots and dicots, respectively. In several organisms such as *Brachypodium dystachon, T. aestivum, Zea mays, Hevea brasiliensis, Populus trichocarpa*, *Malus* × *domestica*, *Prunus persica, Pyrus* × *bretschneideri* and *Solanum lycopersicum*, additional JAZ genes were identified (Supplementary Table S1).

The number of JAZ genes ranged from 1 to 50 in *M. polymorpha* and *T. aestivum*, respectively. However, 10–20 was the most common number of genes in most species (Table 1, Supplementary Table S1). In algae lineage, no JAZ sequences were detected (Table 1). The length of amino acidic sequences was highly variable through different plant lineages, but the normal range was between 100 and 300 aa (Supplementary Table S1). The longest JAZ protein corresponding to *Selaginella moellendorffii* showed 1346 aa, and the shortest exhibited 50 aa in *Erythranthe guttata*. These results indicated that in the different lineages and species, the number of genes and the protein length were variable, whereas these sequences retained the characteristic motifs and domains of the JAZ family.

### 2.2. Conserved Motifs and Domains of JAZ Proteins in Land Plants

**Figure 2:**
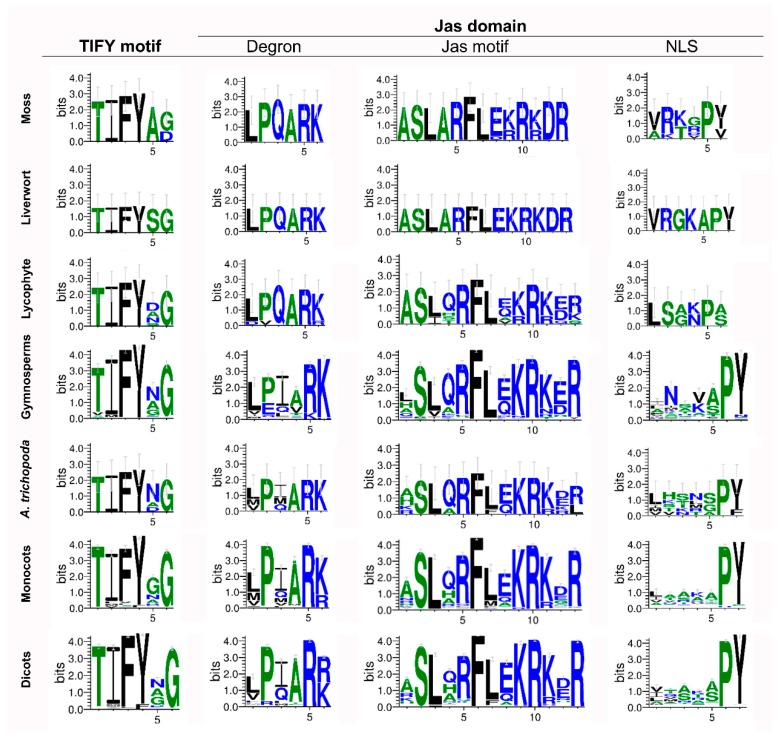
Consensus sequences of the TIFY motif and the Jas domain for different lineages in the plant kingdom. TIFY motif logo sequence and Jas domain showing degron, Jas motif and Nuclear Localization Signal (NLS) logo sequences. Logo sequences of the TIFY motif were obtained from 9, 1, 8, 159, 6, 287 and 515 sequences for moss, liverwort, lycophyte, gymnosperms, *Amborella trichopoda*, monocots and dicots, respectively. Logo sequences of the degron were obtained from 9, 1, 8, 167, 6, 287 and 516 sequences for moss, liverwort, lycophyte, gymnosperms, *A. trichopoda*, monocots and dicots, respectively. Logo sequences of the Jas motif were obtained from 9, 1, 8, 195, 6, 303 and 543 sequences for moss, liverwort, lycophyte, gymnosperms, *A. trichopoda*, monocots and dicots, respectively. Logo sequences of NLS were obtained from 7, 1, 2, 168, 6, 278 and 510 sequences for moss, liverwort, lycophyte, gymnosperms, *A. trichopoda*, monocots and dicots, respectively. JAZ proteins lacking the TIFY motif were not included in the analysis. For more details, see the Materials and Methods section.

To gain insights into the evolution of domains and motifs in lineages of the plant kingdom, consensus sequences of the Jas and CMID domains and the TIFY and EAR motifs were constructed. The TIFY motif and the Jas domain showed stronger conservation in entire plant lineages. However, not all JAZ proteins maintained these sequences (Figure 2, Supplementary Table S1). The majority of JAZ proteins displayed the TIFYXG consensus sequences along the evolutionary history of the plant kingdom (Figure 2, Supplementary Table S1). Motifs of the Jas domain also indicated a high conservation in plant lineages (Figure 2). On one hand, the degron showed a LPQARK conserved sequence in lower plants (liverwort, moss and lycophyte) and LPIAR(R/K) in gymnosperms, *A. trichopoda*, monocots and dicots (Figure 2, Supplementary Table S1). The fifth and sixth amino acidic residues of the degron consensus sequence exhibited the highest conservation (Figure 2). On the other hand, logo sequences of the Jas motif showed higher similarity among lower plants than among higher plants (Figure 2). However, consensus sequences of the Jas motif could be defined as X-SL-X-R-FL-X-KRK-X-R. Finally, Nuclear Localization Signal (NLS) was the most variable motif between different species (Figure 2), defined by the consensus sequence X5-PY [25]. Moreover, the first five amino acidic residues displayed a stronger variability from gymnosperms to dicots (Figure 2, Supplementary Table S1). In the cases of moss and lycophyte, the fifth position was occupied by Pro amino acid (Figure 2, Supplementary Table S1). Therefore, the TIFY motif and the Jas domain were highly conserved in plant kingdom.

**Figure 3:**
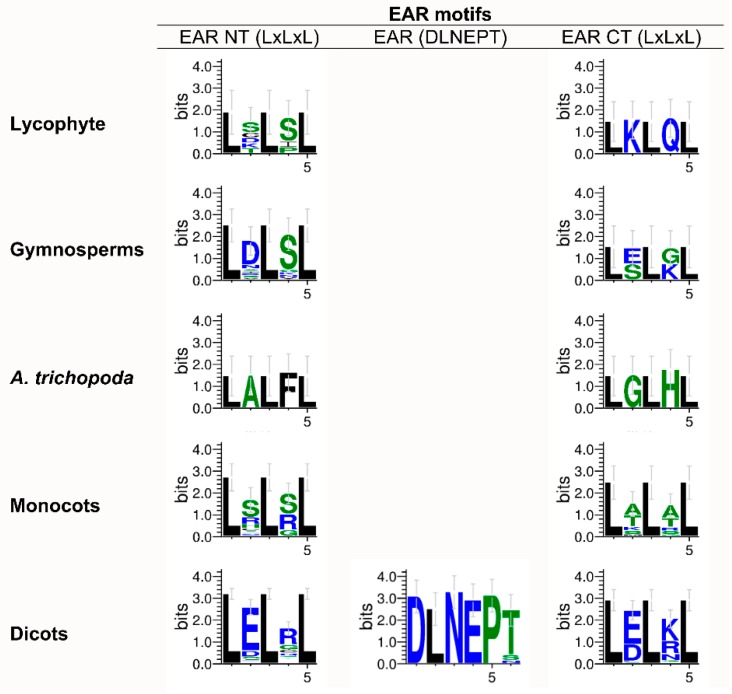
Consensus sequences of EAR motifs for different lineages in the plant kingdom. Logo sequences of the EAR NT motif were obtained from 5, 17, 1, 28 and 118 sequences for lycophyte, gymnosperms, *Amborella trichopoda*, monocots and dicots, respectively. Logo sequences of the EAR (DLNEPT) motif were obtained from 19 sequences for dicots. Logo sequences of the EAR CT motif were obtained from 1, 2, 2, 27 and 45 sequences for lycophyte, gymnosperms, *A. trichopoda*, monocots and dicots, respectively. CT, C-terminal region; EAR, ethylene-responsive element binding factor-associated amphiphilic repression; NT, N-terminal region. For more details, see the Materials and Methods section.

Otherwise, EAR motifs corresponding to both N- and C-terminal regions of JAZ proteins shared the basic sequence LxLxL in all plant lineages (Figure 3). However, the EAR motif defined as DLNEPT, which is located between the TIFY and Jas domains, was only conserved in dicot plants (Figure 3). So, both EAR N- and C-terminals were conserved from moss to dicots, while the EAR motif defined as DLNEPT appeared in dicot plants.

**Figure 4:**
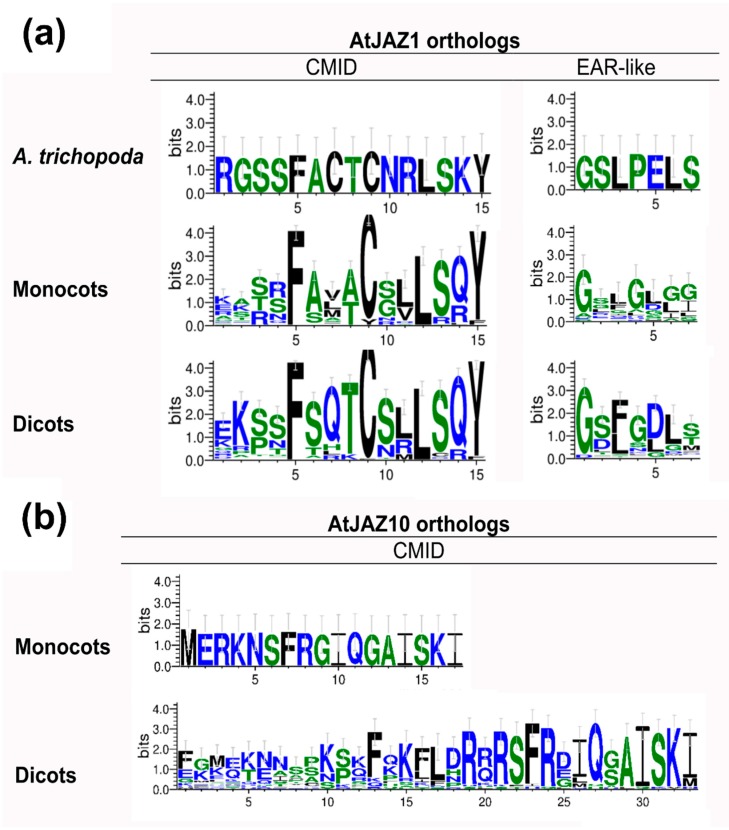
Consensus sequences of the N-terminal region for AtJAZ1 and AtJAZ10 orthologs in different plant kingdom lineages. (**a**) CMID domain and EAR-like logo sequences for AtJAZ1 orthologs in *Amborella trichopoda*, monocots and dicots. (**b**) CMID domain for AtJAZ10 orthologs in monocots and dicots. Logo sequences of the CMID domain were obtained from 1, 55 and 78 sequences for AtJAZ1 orthologs in *A. trichopoda*, monocots and dicots, respectively. Logo sequences of the EAR-like domain for AtJAZ1 orthologs were obtained from 1, 26 and 74 sequences for AtJAZ1 orthologs in *A. trichopoda*, monocots and dicots, respectively. Logo sequences of the CMID domain were obtained from 1 and 28 sequences for AtJAZ10 orthologs in monocots and dicots, respectively. CMID, cryptic MYC2-interacting domain; EAR, ethylene-responsive element binding factor-associated amphiphilic repression; JAZ, jasmonate-ZIM domain. For more details, see the Materials and Methods section.

For a better understanding of the evolution corresponding to the N-terminal region of AtJAZ1 and AtJAZ10 orthologs involved with MYC2 interaction [27], a search was performed on 1065 JAZ protein sequences reported in this research (Supplementary Table S1). Regarding AtJAZ1 proteins, which contain the CMID domain and the adjacent EAR-like motif, gymnosperms and lower plants did not show this conserved region, but it appeared in *A. trichopoda* for the first time, and it was also conserved in angiosperms (Figure 4a). In all groups, the residues F, C, L and Y were the most conserved in the CMID domain. In the case of EAR-Like motif, it displayed a lower conservation in monocots and dicots (Figure 4a). On the other hand, the CMID domain of AtJAZ10 and its orthologs appeared only in the monocot *Elaeis guinensis* (Supplementary Table S1). However, this domain was extensively conserved in dicots (Figure 4b, Supplementary Table S1). In monocots, the CMID domain of the AtJAZ10 ortholog protein was shorter than in dicots, where the monocot had 17 aa against the 33 aa of the dicots (Figure 4b). Besides, the C-terminal region of the CMID domain was more conserved than the N-terminal region in dicots (Figure 4b). Hence, these results showed that the CMID domain of AtJAZ1 and AtJAZ10 orthologs emerged in basal angiosperms and in angiosperms, respectively.

It is important to highlight that some previously reported JAZ proteins showed additional domains by using the Conserved Domains Database (CDD). For instance, the wheat JAZs TaJAZ4-A, TaJAZ4-B, TaJAZ4-D, TaJAZ5-A, TaJAZ11-A, TaJAZ14-A, TaJAZ14-B and TaJAZ14-D [20] exhibited the ZML domain. In any case, these sequences were considered for subsequent analysis. 

### 2.3. Degron Analysis in Land Plants

**Figure 5:**
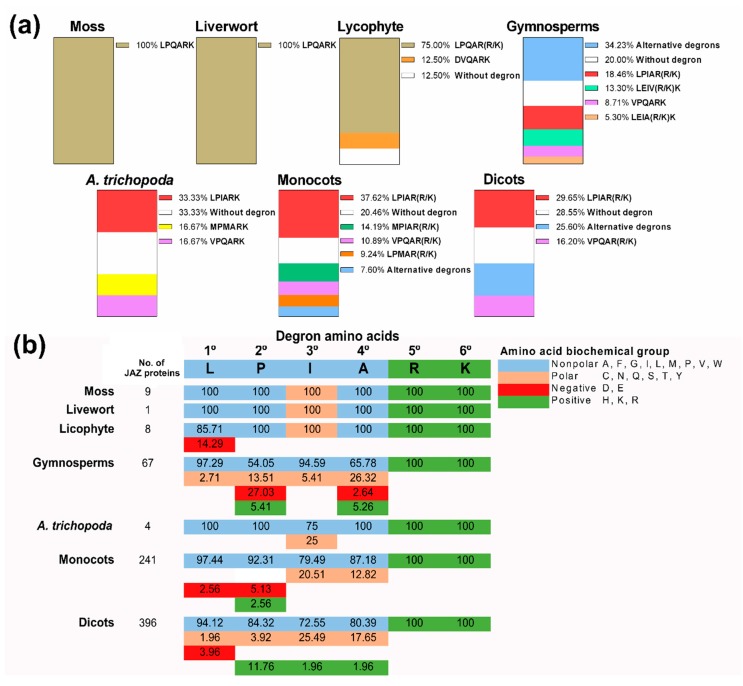
Analysis of degron sequences in different lineages of the plant kingdom. (**a**) Percentage (%) of specific degron sequences in each plant lineage. (**b**) Percentage (%) of different amino acid biochemical groups in each position of the degron sequence compared with the canonical degron LPIARK. The percentage (%) of specific degrons was obtained from 9, 1, 8, 195, 6, 303 and 543 sequences for moss, livewort, lycophyte, gymnosperms, *Amborella trichopoda*, monocots and dicots, respectively. The percentage (%) of different amino acid biochemical groups in each position of the degron sequence was obtained from 1, 1, 3, 37, 3, 39 and 51 different degron sequences for moss, livewort, lycophyte, gymnosperms, *A. trichopoda*, monocots and dicots, respectively.

To explore different degron sequences in land plants, the canonical degron LPIAR(R/K) of *A. thaliana* was used as a reference because it is a critical motif for the co-receptor COI1–JAZ complex formation. We took into account that the amino acidic residues at the fifth and sixth position are the most important for the degron functionality [3,45], through all JAZ proteins in the plant kingdom. Next, we searched degron variants in entire species according to these criteria. As we moved through the evolutionary scale, from moss to dicots, we observed that the number of degron variants increased (Figure 5a, Supplementary Table S2). Moss and liverwort shared 100% frequency of the degron sequence LPQARK (Figure 5a, Supplementary Table S2). In lycophyte, LPQARK was the major degron and for first time, the new degron sequence DVQARK appeared (Figure 5a, Supplementary Table S2). In the case of gymnosperms, the majority of sequences displayed alternative degrons (Supplementary Table S2), and the canonical degron LPIAR(R/K) represented 18.46% (Figure 5a). Also, LEIV(R/K)K, VPQARK and LEIA(R/K)K degrons surged in this group (Figure 5a). Otherwise, the basal angiosperm *A. trichopoda* exhibited LPIARK with higher frequency, as well as VPQARK for gymnosperms (Figure 5a). We considered that monocots and dicots contained a similar percentage of the canonical degron LPIAR(R/K), and in these groups, the diversity of alternative degrons increased (Figure 5a). Finally, from lycophyte to dicots, JAZ sequences without degrons emerged (Figure 5a). Thus, the canonical sequence LPIAR(R/K) was the most represented degron from gymnosperms to dicots. However, a wide diversity of degron sequences was observed for gymnosperm, monocot and dicot lineages (Figure 5a). Besides this, JAZ proteins without degron sequences emerged in lycophytes and were maintained to dicots (Figure 5a).

To study the conservation of the amino acid biochemical features in degrons, we analyzed the percentage of each biochemical group at the different positions in degron sequences containing Arg/Lys (R/K) at the fifth and sixth positions, respectively. We observed differences depending on plant lineage. For instance, degrons in moss and liverwort maintained the same pattern of biochemical groups at the first four amino acid positions: the first, second and fourth positions corresponded to non-polar amino acids, and the third corresponded to amino acids with polar groups in their lateral chain (Figure 5b). In lycophyte, the first amino acid can also be a negatively charged amino acid (Figure 5b). Gymnosperms’ degron was characterized by polar and non-polar amino acids at the first and third positions, while any biochemical group was present at the second and fourth positions (Figure 5b). In *A. trichopoda*, non-polar amino acids were established at the first four positions and the third position can be occupied by polar residues (Figure 5b). In monocots and dicots, non-polar residues appeared mostly at the first four positions. However, depending on the lineage, the resting positions showed higher variability (Figure 5b). Therefore, as we moved along the evolutionary scale of the plant kingdom, a diversity of amino acidic residues with different physicochemical characteristics at the first four positions of the degron sequence was observed.

### 2.4. Phylogenetic Analysis of JAZ Proteins in Land Plants

**Figure 6:**
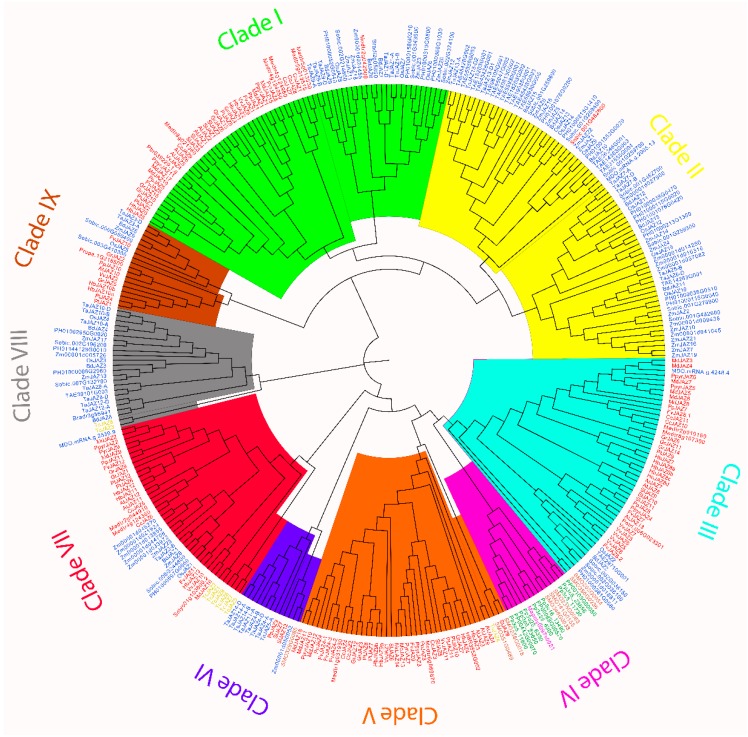
Phylogenetic analysis of JAZ proteins in land plants. The phylogenetic tree was constructed by using the full-length amino acid sequences of liverwort, moss, lycophyte, gymnosperms, monocots and dicots. Purple, green, brown, yellow, blue and red used in sequence names indicate liverwort, moss, lycophyte, gymnosperm, monocot and dicot JAZ proteins, respectively. Different colors indicate clades for different JAZ ortholog proteins. JAZ, jasmonate-ZIM domain.

In order to study the phylogenetic relationships between different JAZ protein orthologs in different plant lineages, an unrooted phylogenetic tree was constructed by the Maximum Likelihood method. Globally, JAZ ortholog proteins were grouped in nine (I–IX) clades (Figure 6). *A. thaliana* JAZ1, JAZ2, JAZ5 and JAZ6 were grouped together in clade I, which contained ortholog sequences from monocots and dicots (Figure 6). Clade II was only constituted by JAZ orthologs of monocots and a JAZ ortholog of *M.* × *domestica* (Figure 6). Otherwise, clade III clustered AtJAZ7, AtJAZ8 and AtJAZ13 ortholog proteins in dicots, but also showed grouping along with JAZ ortholog proteins of some monocot species (Figure 6). In clade IV, JAZ proteins corresponding to liverwort, moss and lycophyte were grouped together (Figure 6). AtJAZ3, AtJAZ4 and AtJAZ9 were clustered near their ortholog proteins with some JAZ proteins of lycophyte and gymnosperms into clade V (Figure 6). On the other hand, clade VI included some JAZ proteins of dicots and monocots along with a lycophyte JAZ protein (Figure 6). Clade VII showed the clustering of AtJAZ11, AtJAZ12 and their respective orthologs in gymnosperms, monocots and dicots. Moreover, clade VIII only clustered gymnosperm and monocot JAZ sequences. Finally, AtJAZ10 orthologs were grouped in clade IX (Figure 6). These results indicated that different JAZ ortholog proteins are phylogenetically related based on their higher similarity and land plant lineage.

### 2.5. Ancestral Sequences of JAZ Proteins in Land Plants

**Figure 7:**
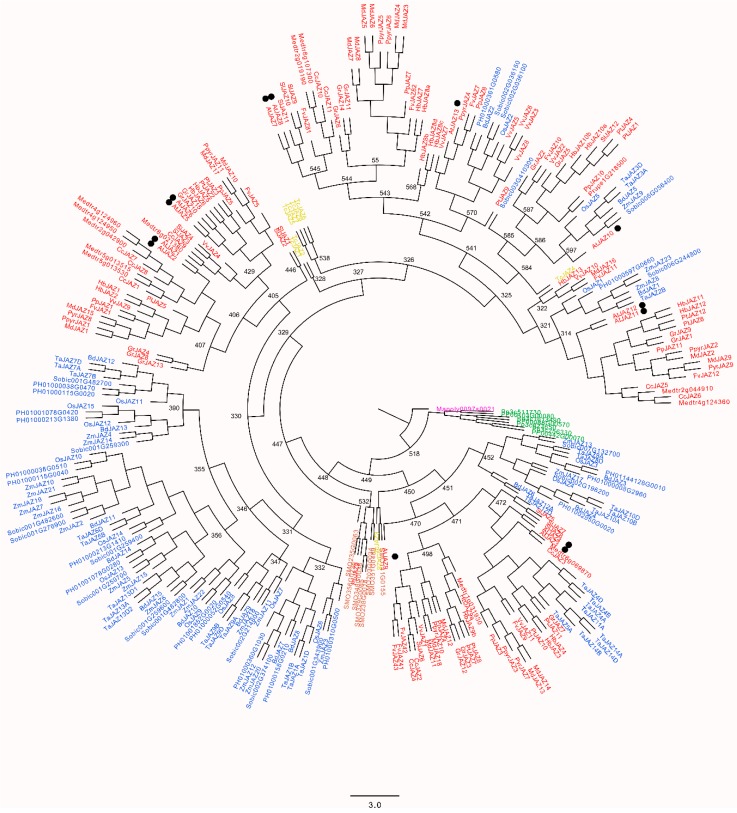
Reconstruction of JAZ ancestral sequences in land plants. The ancestral tree was performed using 308 full-length sequences corresponding to liverwort, moss, lycophyte, gymnosperms, monocots and dicots. Node numbers indicate some output ancestors obtained by using the PhyloBot software. The analysis of JAZ ancestral sequences is available to download at the following URL: http://www.phylobot.com/464456268 (last accessed date: 19 August 2019).

To understand the evolution of the JAZ protein family, a reconstruction of the JAZ ancestral sequences from liverwort to dicots was performed by the Maximum Likelihood method. For this analysis, only 308 JAZ sequences were considered, according to previously characterized JAZ sequences, including additional putative JAZs reported in this research (Table 1). As a result of this analysis, an ancestral tree grouping all JAZ proteins was obtained. The PhyloBot software showed a set of ancestors represented by numbers (Figure 7, Supplementary Table S3, http://www.phylobot.com/464456268, last accessed date: 19 August 2019). The JAZ protein of *M. polymorpha* was individually grouped in the first tree branch. Next, the ancestor 518 was common from moss to dicots (Figure 7). Then, a series of ancestors originated different tree branches, which clustered different species of lycophyte, gymnosperms, monocots and dicots. First, AtJAZ3, AtJAZ4 and AtJAZ9 orthologs diverged in an independent manner from the common ancestor 450 (Figure 7). AtJAZ9 appeared first, and then AtJAZ3 and AtJAZ4 ortholog proteins diverged. The *Fragaria vesca* JAZs FvJAZ4-1, FvJAZ4-2, FvJAZ4-3 and FvJAZ9 were originated from the ancestor 471 (Figure 7). Second, the ancestor 329 and subsequent ancestors originated the groups of JAZ1, JAZ2, JAZ5 and JAZ6 orthologs. AtJAZ1, AtJAZ2 and FvJAZ1 arose from the ancestor 406, which was also the ancestor sequence of AtJAZ5, AtJAZ6 and FvJAZ5. An independent branch of the ancestor 330 gave rise to an exclusive group of monocot JAZ sequences (Figure 7). Third, the ancestors 327 and 326 originated some *Taxus chinensis* JAZ proteins. Fourth, the ancestors 325 and 541 were the common ancestors for AtJAZ10, AtJAZ7, AtJAZ8 and AtJAZ13 orthologs, respectively. These orthologs shared the ancestor sequence 541 and were separated in two subgroups. Fourth, the ancestor sequence 321 initiated the cluster of AtJAZ11 and AtJAZ12 orthologs (Figure 7). Finally, some ancestors, such as 346 and 406, were exclusively ancestors of JAZs for monocot and dicot plants, respectively. These results displayed an approach to the evolutionary history of different JAZ subgroups.

## 3. Discussion

JAZ proteins are key positive and negative regulators of the JA-signaling pathway [46], regulating different JA-responses such as tolerance against biotic and abiotic stresses, developmental processes, reproduction and secondary metabolism [29,47]. However, the evolutionary history of JAZ proteins and how new functionalities emerged along with the plant kingdom evolution are still unclear. In this research, the changes in JAZ protein sequences along the evolutionary scale of land plants are reported. Moreover, the ancestral sequences that gave rise to different JAZ groups are proposed by the reconstruction of JAZ ancestors.

The mechanism of JA-signal transduction is similar to that of auxins, which contain a receptor, the ubiquitin ligase complex for repressors’ degradation, co-repressors, specific transcription factors and homologous repressors, leading to the hypothesis that JAs and auxins share a common ancestor [2]. The JA-signaling pathway, including JAZ proteins, is conserved in liverwort, moss, lycophyte, monocots and dicots [31]. However, JAZ protein members are more numerous in dicots versus liverwort [14,15,31], according to what was observed in the 82 organisms considered in this study (Table 1). The number of JAZ proteins increased during the evolution of vascular plants (Table 1), and this possibly boosted the colonization of new environments, enabling a higher tolerance against stress [1]. However, this expansion of the JAZ gene family was not related to an increase in gene number (Table 1), similar to that observed for the JAZ-homologous repressors, AUX/IAA proteins [48]. A high number of JAZ members involves functional redundancy, such as that reported in Arabidopsis [29] against what was observed in *M. polymorpha,* which only contains a single JAZ protein [14,15]. The expansion of JAZ proteins may be the consequence of gene duplication, alternative splicing and the emergence of new functional domains in proteins [27,49,50].

JAZ proteins in Arabidopsis and in other organisms such as *O. sativa, T. aestivum, S. lycopersicum* or *F. vesca* shape different groups regarding the conserved domains [17,18,19,20]. JAZ proteins, a subfamily within the TIFY family, are characterized by the TIFY domain [21,23] and the Jas domain [21,25]. In Arabidopsis, AtJAZ1–12 contain both conserved domains, while AtJAZ13 is non-TIFY domain [17,22]. JAZ proteins in non-vascular plants exhibited TIFY and Jas domains, but the gymnosperms showed some sequences without the TIFY domain (Supplementary Table S1), suggesting that this domain arose in the ancestor of this lineage. It is noteworthy that the TIFY and Jas domains were highly conserved from liverwort to dicots (Figure 2), according to what was previously reported in different species [17,18,19,20,41]. However, some repressors such as AtJAZ7, AtJAZ8 and AtJAZ13 contain an alternative degenerated Jas domain, which was observed from gymnosperms to dicots, ruling out the perception of JA-Ile and JAZ degradation [22,26]. Additional protein regions, such as EAR motifs, are conserved in AtJAZ5, AtJAZ6, AtJAZ7 and AtJAZ8 [26]. EAR motifs are involved in the recruiting of TPL proteins, which are present in land plants and in algae lineage [2,31], and proteins containing these motifs were detected in all plant lineages from moss to dicots (Figure 3). This suggests that JAZ proteins evolved from an ancestor sequence with EAR motifs. Otherwise, the CMID domain, involved in the interaction between JAZ repressors and MYC2 transcription factors, is weakly conserved through evolution and is different between AtJAZ1 and AtJAZ10 orthologs [27]. Although the logo sequences indicated a high conservation of this domain in monocots and dicots (Figure 4), the evolution for each one seems to be different because the amino acid composition is slightly variable and CMID is only present in AtJAZ10 orthologs of monocots and dicots, while for AtJAZ1 orthologs, it is represented from basal angiosperms (Figure 4). 

The degron sequence of JAZ repressors is related with the JAZ degradation by the 26S proteasome after the formation of the complex JAZ–JA-Ile–COI1 [3], promoting the release of MYC2 TFs and the activation of JA responses [5,6]. The canonical degron LPIAR(R/K) was described in *A. thaliana* JAZ proteins for the first time, and then other species showed this conserved sequence (Supplementary Table S1) [17,18,19,30]. We also observed that the LPIAR(R/K) sequence is the most common degron, at least for gymnosperms, *A. trichopoda*, monocots and dicots (Figure 5a). In contrast, JAZ proteins of lower plants such as moss and lycophyte exhibited LPQARK (Figure 5a), the same sequence reported for the liverwort *M. polymorpha* [14,15]. The last amino acidic residues R and R/K are directly involved in the interaction with COI1 and JA-Ile, unlike the other residues that could participate in the three-dimensional structure and the binding affinity to the ligand, therefore regulating this interaction [3]. During the evolution of land plants, we observed that the number of alternative degrons rises, changing in the first four residues (Figure 5a). It is important to note that some JAZ proteins lacking the conserved degron emerged in the lycophyte lineage, according to that reported for the AtJAZ7, AtJAZ8 and AtJAZ13 orthologs [5,22]. On the other hand, the physicochemical characteristics of the different amino acidic residues are critical for the structure and function of the proteins [51,52]. We observed that all of the JAZ proteins containing the conserved degron maintained R and R/K at the fifth and sixth positions respectively (Figure 5b), as reported in Sheard et al. [3], so the function is determined by their physicochemical properties. This could be applied to the first four residues, detecting that in different lineages these positions can be occupied by different amino acids with similar physicochemical groups. Otherwise, the change of the fifth positively charged residues prevents the interaction with COI1 [26]. In summary, although the sequence LPIAR(R/K) is the canonical degron, some JAZ proteins changed to amino acid residues with similar or different biochemical properties, extending the diversity of degrons in the plant kingdom.

Therefore, all ortholog proteins belonging to different JAZ families in land plants showed similar conserved domains, and this is evidenced in the phylogenetic relationships. The phylogenetic tree and JAZ ancestral sequence reconstruction included previously reported and new putative JAZ sequences identified in the present research (Table 1). The more similar JAZ sequences are clustered closely (Figure 6), according to what was previously observed in different plant species such as *S. lycopersicum* [17], *V. vinifera* [30], *F. vesca* [18], *O. sativa* [19], and *T. aestivum* [20]. Specifically, AtJAZ1 and AtJAZ2 ortholog proteins clustered in the same subgroup (Figure 6), similar to that observed in *O. sativa* [19], *F. vesca* [18], and *M.* × *domestica* [41]. Otherwise, AtJAZ5 and AtJAZ6 orthologs, which contain a conserved EAR motif in the C-terminal region (Supplementary Table S1) [28], are grouped in the same clade (Figure 6), according to what was observed in *F. vesca* [18]. AtJAZ3, AtJAZ4 and AtJAZ9 proteins contain highly similar sequences and shape the same clade [17,18,41], in a comparable manner to what was observed in this research (Figure 6). Moreover, AtJAZ10 orthologs showing the CMID domain in a single monocot and in several dicots (Figure 4b, Supplementary Table S1), according to previous reports for Arabidopsis [27] and FvJAZ10 [18], formed an independent clade, as well as AtJAZ11 and AtJA12 ortholog proteins (Figure 6). Finally, a well differentiated clade is formed by JAZ lacking the conserved Jas (AtJAZ7 and AtJAZ8) and TIFY (AtJAZ13) domains. So, phylogenetic trees allow us to relate ortholog JAZ proteins regarding their sequence similarities and domain conservation. However, how the different JAZ protein subgroups evolved from an ancestral JAZ sequence in land plants cannot be elucidated.

The reconstruction of the ancestral sequence for a specific protein family is a powerful tool to understand the origin and the evolutionary process followed by protein sequences [53], but the current software can only provide the most conserved domains with higher fidelity [54]. JAZ proteins are represented in lower land plants by 1–9 members (Table 1, Supplementary Table S1) [14,15,21], compared to 40, 50 and 13 members in *Pinus taeda, Zea mays* and *A. thaliana*, respectively (Table 1) [22], showing a big expansion of the JAZ repertoire in vascular plants. Until now, *M. polymorpha* is the least evolved land plant with a sequenced genome [55], and its single JAZ protein has the TIFY and Jas domains highly conserved (Supplementary Table S1, Figure 2) [14,15], so it is the best model to study the evolution and functions of JAZ proteins [46]. The liverwort lineage is located in the first branch of the ancestral tree (Figure 7), suggesting that an ancestral sequence would be the origin of JAZ proteins. Being that the algae lineage contains TPL proteins [31], which are recruited by EAR motifs such as LxLxL [9], we suppose that the JAZ ancestor had this motif. However, because of proteins in lower plants, the conservation of these motifs is not represented in the ancestral sequences. In the reconstruction of the evolutionary history of JAZ ancestors, the other JAZ proteins in the different lineages were originated from the ancestor 518 (Figure 7). The group of the AtJAZ3, AtJAZ4 and AtJAZ9 ortholog proteins experienced a divergence from the ancestor 449, constituting a well-defined group (Figure 7). Then, a set of ancestral JAZ proteins gave rise to the ancestor 330, which was separated into two branches: AtJAZ1, AtJAZ2, AtJAZ5 and AtJAZ6 orthologs grouping dicots in the same clade, while monocots were grouped in the other JAZ ortholog group (Figure 7). AtJAZ5 and AtJAZ6 contain the conserved motif LxLxL [28], while AtJAZ1 and AtJAZ2 lack it, suggesting that the ancestor 406 originator owned this conserved motif, but this one was lost during the evolution of AtJAZ1 and AtJAZ2 orthologs and maintained to the AtJAZ5 and AtJAZ6 origin (Figure 7). The last big group of JAZ orthologs showed the sequence 327 as the common ancestor for AtJAZ7, AtJAZ8, AtJAZ13, AtJAZ10, AtJAZ11 and AtJAZ12 (Figure 7), which diverged into three differentiated groups: the first containing AtJAZ7, AtJAZ8, and AtJAZ13, the second only with AtJAZ10, and the third with AtJAZ11 and AtJAZ12. These groups are characterized by the lack of the degron sequence for COI1 interaction in AtJAZ7, AtJAZ8 and AtJAZ13 [22,26], and the presence of the CMID conserved domain in the case of the AtJAZ10 orthologs [27]. An important point is that AtJAZ7 and AtJAZ8 ortholog proteins contain the EAR motif in the N-terminal region [18,26], suggesting that the previous ancestors should have contained this conserved sequence. In summary, the oldest ancestor would show the TIFY and Jas domains according to what was observed in the JAZ sequence of liverwort (Figure 7) [14,15].

**Figure 8:**
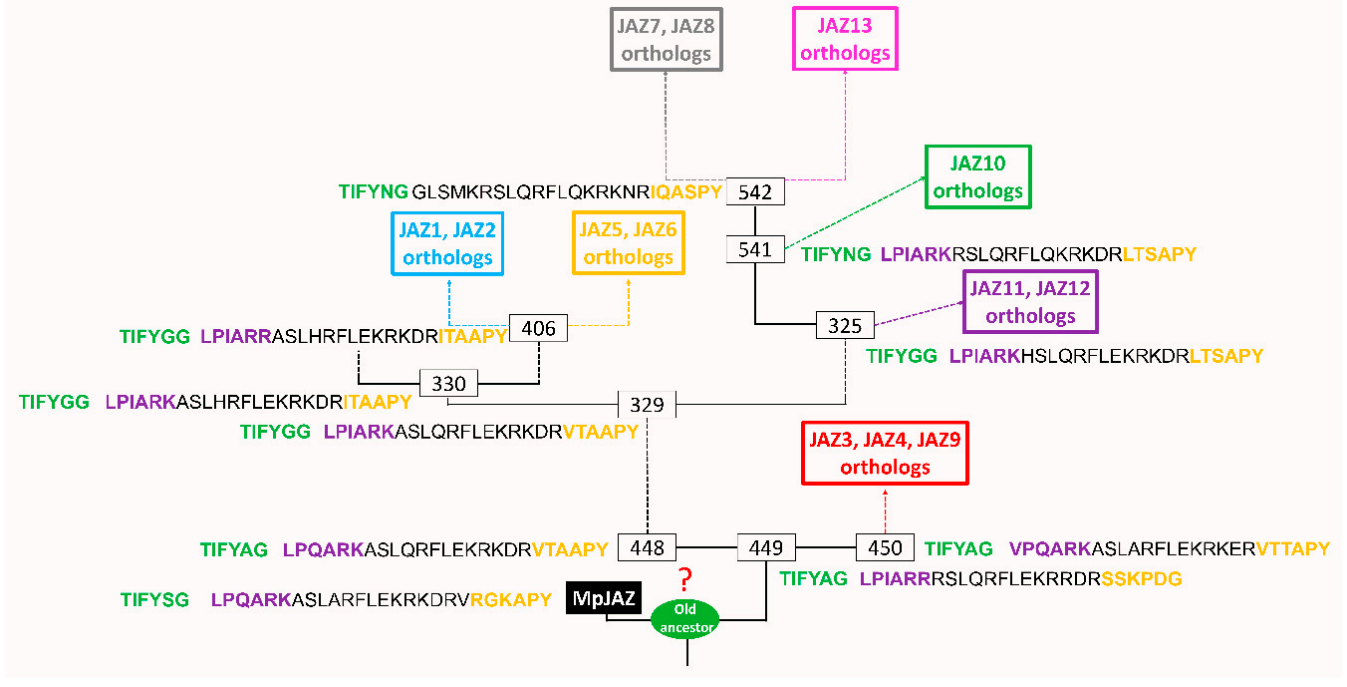
Proposed model of JAZ protein evolution. Numbers framed in boxes indicate the output name of the ancestor assigned by the PhyloBot software. The analysis of JAZ ancestral sequences is available to download at the following URL: http://www.phylobot.com/464456268, last accessed date: 19 August 2019. The TIFY motif and the Jas domain are displayed near to each ancestor. Different colors in boxes show different subgroups of eight JAZ ortholog proteins. Dashed lines indicate intermediate ancestors. The length of branches does not correspond to evolution time. JAZ, jasmonate-ZIM domain.

Subsequently, we propose a general model of JAZ evolution in land plants, showing changes in ancestral sequences in eight different JAZ groups (Figure 8). During the evolutionary process, the putative ancestors (Supplementary Table S3) showed small changes in amino acidic residues of the TIFY and Jas domains, allowing the evolution of structures and functions. The TIFY motif is the most conserved sequence through evolution. In the case of the degron and Jas motif, some synonym substitutions are observed between the different ancestors (Figure 8), so this may possibly involve an adaptation to the environment [46]. It is noteworthy that the AtJAZ7, AtJAZ8 and AtJAZ13 orthologs’ clade lost the Jas conserved motif during evolution, originating a new functional group of JAZ proteins. Besides that, the non-TIFY JAZ13 repressors lost the TIFY domain (Figure 7 and Figure 8). Otherwise, the NLS region is the most variable between different JAZ groups, similar to what was observed for AUX/IAA proteins [48]. However, the oldest ancestor of the JAZ proteins of land plants is still unknown.

Regarding physiological implications, JAs are essential signaling phytohormones for stress tolerance, metabolism and developmental responses [29]. Thus, the evolution of key components of the signaling pathway, such as JAZ repressors, is necessary for overcoming environmental constraints [46]. In this sense, JAZ proteins are critical for the activation of JA responses, through the perception mechanism of JA-Ile and dinor-OPDA in *A. thaliana* and *M. polymorpha*, respectively [3,14]. The degron sequence is critical for interaction with COI1 and JA-Ile in *A. thaliana* [3], and a high diversity of degron sequences was detected in land plants (Supplementary Table S2, Figure 5). This could be related to different interaction affinities for the perception complex, because JAZs containing different degron sequences show diverse binding affinities in the interaction with COI1 mediated by JA-Ile [26], leading to fine-tuning of JA-response activation depending on the type of JAZ protein involved. In the case of JAZ7, JAZ8 and JAZ13 orthologs, they lack the conserved degron sequence (Supplementary Table S1) [22,26] and this prevents their degradation and maintains the repression over the transcription factors, which is related to pulsed responses and desensitization to JAs [5]. Besides this, JAZ proteins are involved in the formation of a co-repressor complex by the interaction with NINJA adaptor protein [27], and they recruit TPL proteins mediated by TIFY and EAR-like motifs, respectively [25]. Thus, JAZ5–8 and JAZ13 orthologs that contain EAR motifs in land plants (Supplementary Table S1, Figure 3) allow the formation of a repressor complex and the inhibition of JAZ responses mediated by TFs [22,26]. Some JAZ orthologs such as JAZ1 and JAZ10 show the N-terminal region containing the CMID domain and the EAR-like motif, involved in the attenuation of JA responses by the repression of MYC2 TF [27]. Moreover, JAZs are repressors of different TF families involved in defense, secondary metabolite biosynthesis, and tolerance to abiotic stress, among others [29]. In this case, JAZ proteins show a certain degree of functional redundancy, but it seems to be restrained to specific TF families [29]. The JAZ–TFs interaction is mediated by the Jas motif [25], which is highly conserved along the evolutionary scale (Supplementary Table S1, Figure 2), however, specific interactions are observed between JAZ proteins and some TFs [29]. In summary, the gain or loss of specific domains and motifs, along with changes in the amino acid composition observed in different lineages of land plants (Supplementary Table S1, Figure 1, Figure 2, Figure 3 and Figure 4), may explain the higher ability for adaption under different environmental conditions and stresses.

In conclusion, all lineages of land plants contain JAZ proteins with the TIFY and Jas domains highly conserved. Besides this, some JAZs in subgroups such as JAZ1, JAZ5/6, JAZ7/8, and JAZ10 hold additional domains, e.g., EAR motifs or CMID domains. However, the origin of these domains is unknown, although it could be explained by some still unknown ancestor. New approaches for studying the conservation and evolution of protein sequences in each lineage could clarify the origin of JAZ proteins in the plant kingdom. These results establish a basis to understand the functional role of JAZ proteins during the evolution process in land plants, and they could be used to obtain crops more tolerant to environmental stresses through genetic breeding or gene modification.

## 4. Materials and Methods 

### 4.1. Bioinformatic Identification of JAZ Proteins

JAZ protein sequences of *A. thaliana* were used for the searching of ortholog proteins in different plant genomes available in the PLAZA v4.0 database (Ghent University, Belgium, https://bioinformatics.psb.ugent.be/plaza/, last accessed date: 9 August 2019) by the BLASTP tool. Then, the resulting sequences were filtered. First, partial sequences and those with an e-value > 0.0 were removed. Second, NCBI’s Conserved Domain Database (CDD) was used for the identification of proteins containing the Jas domain, and proteins lacking this sequence were removed. Third, sequences containing PPD or ZML domains were removed. Fourth, redundant sequences were removed after the multiple alignment with those previously reported by using Clustal Omega v2.0.12 (EMBL-EBI, Wellcome Genome Campus, Cambridgeshire, UK, https://www.ebi.ac.uk/Tools/msa/clustalo/, last accessed date: 15 April 2019). The resulting 1065 JAZ sequences used in the following analysis can be found in Supplementary Table S1.

### 4.2. Structural Analysis of JAZ Protein Domains

The TIFY and Jas domains of newly identified JAZ proteins in moss, lycophytes, gymnosperms, *A. trichopoda*, monocots and dicots were characterized by multiple alignment with respect to *A. thaliana* JAZ proteins and by using the Conserved Domains Database (CDD) v3.12 (National Center of Biotechnology Information, Maryland, USA; https://www.ncbi.nlm.nih.gov/Structure/cdd/cdd.shtml, last accessed date: 17 April 2019). Other conserved sequences, such as CMID domains and EARs motifs, were searched manually using *A. thaliana* sequences as a reference. The TIFY and Jas domains of previously reported JAZ proteins were obtained from the information collected in relative publications (Supplementary Table S1). Logo sequences of structural domains were obtained by the Weblogo v3.0 tool (University of California, Berkeley, California, USA; http://weblogo.threeplusone.com/create.cgi, last accessed date: 7 May 2019).

### 4.3. Analysis of Degron Sequences

For the degron analysis in the different plant lineages, only amino acidic sequences with Arg/Lys (R/K) at the fifth and sixth amino acidic positions from N- to C-terminus according to the canonical degron sequence LPIAR(R/K) [3] were considered as functional degrons. Then, the percentage (%) of each specific degron was calculated in the different lineages. In order to clearly display the more represented degrons, all sequences that individually showed a percentage lower than 5% were included together as alternative degrons. The percentage of each type of amino acidic residue at the different positions was calculated according to the physicochemical properties (non-polar, polar, negatively or positively charged) by using the Amino Acid Explorer tool (University of Maryland, Maryland, USA; https://www.ncbi.nlm.nih.gov/Class/Structure/aa/aa_explorer.cgi, last accessed date: 12 June 2019).

### 4.4. Phylogenetic Analysis

The evolutionary relationships between JAZ proteins were inferred using 308 full-length amino acidic sequences by using the Maximum Likelihood algorithm and a Jones, Taylor and Thornton (JTT) matrix-based model. The bootstrap consensus trees were inferred from 1000 replicates. Evolutionary analyses were conducted in Molecular Evolutionary Genetics Analysis X (MEGA X) (University of Pennsylvania, Pennsylvania, USA [56], last accessed date: 9 August 2019). An unrooted phylogenetic tree was constructed using 308 amino acidic JAZ sequences corresponding to previously identified and characterized JAZ proteins.

### 4.5. Reconstruction of JAZ Ancestral Sequences

For the ancestral JAZ reconstructions, different AtJAZ protein orthologs from different species were used. The amino acidic sequences were obtained from the PLAZA v4.0 database (Ghent University, Belgium, https://bioinformatics.psb.ugent.be/plaza/, last accessed date: 7 August 2019) and from previously reported JAZ sequences. Ancestral sequences were inferred using PhyloBot software (University of California, San Francisco, USA) [57] and using 308 JAZ sequences. Sequences were aligned by using MSAProbs [58] and MUltiple Sequence Comparison by Log-Expectation (MUSCLE) (California, USA) [59] with default settings. The results of the MSAProbs alignment and the tree drawn under the PROTCATJTT model were further analyzed and visualized by FigTree v1.4.3 (The University of Edinburgh, Scotland, UK, http://tree.bio.ed.ac.uk/software/figtree/, last accessed date: 16 August 2019). The analysis of JAZ ancestral sequences is available to download at the following URL: http://www.phylobot.com/464456268 (last accessed date: 19 August 2019).

## Figures and Tables

**Table 1:** JAZ family genes in the plant kingdom.

Lineage	Organism	Number of Genes	Number of JAZ Genes	Reference
Algae	*Chlamydomonas reinhardtii*	17,741	0	[21], this research ^1^
Moss	*Physcomitrella patens*	32,926	9	[21], this research
Liverwort	*Marchantia polymorpha*	19,278	1	[14]
Lycophyte	*Selaginella moellendorffii*	22,285	8	[21]
Gymnosperms	*Cycas micholitzii*	28,901	2	This research
	*Ginkgo biloba*	30,404	7	This research
	*Gnetum montanum*	32,549	2	This research
	*Picea abies*	66,632	30	This research
	*Picea glauca*	28,909	14	This research
	*Picea sitchensis*	20,434	12	This research
	*Pinus pinaster*	76,426	19	This research
	*Pinus sylvestris*	36,106	25	This research
	*Pinus taeda*	84,446	40	This research
	*Pseudotsuga menziesii*	149,717	30	This research
	*Taxus baccata*	32,062	5	This research
	*Taxus chinensis*	Unknown	9	[33]
Amborellales	*Amborella trichopoda*	26,846	6	This research
Monocots	*Spirodela polyrhiza*	19,623	7	This research
	*Elaeis guineensis*	29,808	14	This research
	*Ananas comosus*	270,240	8	This research
	*Musa acuminata*	37,582	34	This research
	*Phalaenopsis equestris*	29,431	9	This research
	*Brachypodium distachyon*	34,310	17	[21,34], this research
	*Hordeum vulgare*	25,780	8	This research
	*Oropetium thomaeum*	28,446	16	This research
	*Oryza brachyantha*	34,155	9	This research
	*Oryza sativa*	42,189	15	[19,21]
	*Phyllostachis edulis*	31,978	18	[35]
	*Setaria italica*	34,584	18	This research
	*Sorghum bicolor*	34,211	18	[21], this research
	*Triticum aestivum*	114,581	50	[20],this research
	*Zoysia japonica*	59,271	18	This research
	*Zea mays*	44,474	38	[36], this research
	*Zostera marina*	20,450	7	This research
Dicots	*Actinidia chinensis*	39,040	5	This research
	*Amaranthus hypochondriacus*	23,847	8	This research
	*Beta vulgaris*	26,920	7	This research
	*Chenopodium quinoa*	44,776	6	This research
	*Daucus carota*	32,113	10	This research
	*Arabidopsis lyrata*	31,073	13	This research
	*Arabidopsis thaliana*	69,810	13	[17,22]
	*Brassica oleraceae*	59,225	25	This research
	*Brassica rapa*	40,492	28	This research
	*Capsella rubella*	26,521	10	This research
	*Schrenkiella parvula*	26,313	12	This research
	*Carica papaya*	27,768	7	This research
	*Tarenaya hassleriana*	30,556	16	This research
	*Citrullus lanatus*	597,261	10	This research
	*Erythranthe guttata*	28,140	10	This research
	*Cucumis melo*	28,608	10	This research
	*Cucumis sativus*	21,503	11	This research
	*Hevea brasiliensis*	42,550	14	[37,38], this research
	*Manihot esculenta*	33,033	16	This research
	*Ricinus communis*	31,221	9	This research
	*Arachis ipaensis*	41,840	10	This research
	*Cajanus cajan*	48,680	11	[39]
	*Cicer arietinum*	170,274	9	This research
	*Glycine max*	56,044	24	This research
	*Medicago truncatula*	50,894	12	[21], this research
	*Trifolium pratense*	39,948	10	This research
	*Vigna radiata*	22,368	10	This research
	*Utricularia gibba*	25,930	11	This research
	*Gossypium raimondi*	37,505	15	[40]
	*Theobroma cacao*	29,232	8	This research
	*Corchorus oliotorus*	37,281	8	This research
	*Eucalyptus grandis*	36,349	11	This research
	*Nelumbo nucifera*	26,685	9	This research
	*Ziziphus jujuba*	31,701	10	This research
	*Fragaria vesca*	32,831	12	[18]
	*Malus* × *domestica*	55,620	21	[41], this research
	*Prunus persica*	26,873	12	[42], this research
	*Pyrus* × *bretschneideri*	42,812	12	[43], this research
	*Coffea canephora*	469,604	7	This research
	*Citrus clementina*	24,533	7	This research
	*Populus trichocarpa*	42,950	13	[44], this research
	*Capsicum annum*	35,884	10	This research
	*Solanum lycopersicum*	34,725	13	[17], this research
	*Solanum tuberosum*	39,028	13	This research
	*Petunia axillaris*	35,812	13	This research
	*Vitis vinifera*	26,346	11	[21,30]

^1^ ‘This research’ refers to new JAZ sequences found in the PLAZA 4.0 database. For more details, see the Materials and Methods section.

## Supplementary Materials

Supplementary materials can be found at https://www.mdpi.com/1422-0067/20/20/5060/s1. Table S1. Main amino acid sequence features of JAZ proteins of different plant lineages contained in the PLAZA database. Table S2. List of specific degron sequences for different plant lineages. Table S3. List of protein ancestor sequences obtained by PhyloBot software.
