# Supplementary material for: Evolutionary Analysis of JAZ Proteins in Plants: An Approach in Search of the Ancestral Sequence
Source: Int J Mol Sci. 2019 Oct 12;20(20):5060. doi: 10.3390/ijms20205060 (PMC6829463; doi:10.3390/ijms20205060)
Supplement: Supplementary file 1 [file ijms-20-05060-s001.zip › Table S2.docx]

**Table S2.** List of specific degron sequences for different plant lineages.

| **Moss** | **Liverwort** | **Lycophyte** | **Gymnosperms** | ***Amborella trichopoda*** | **Monocots** | **Dicots** |
| --- | --- | --- | --- | --- | --- | --- |
| LPQARK | LPQARK | DVQARK  LPQARR  LPQARK | FPIARK | LPIARK | AIQARR | APQARK |
|  |  | LPQARR | FPLQKR | MPMARK | FPLARR | DRIARR |
|  |  | LPQARK | IPIARK | VPQARK | IPIARK | ERIARR |
|  |  |  | IPIARR |  | IPLSRK | FPIARK |
|  |  |  | IPISRK |  | IPLVRR | FPIARR |
|  |  |  | IPLARK |  | IPQARK | FPITRR |
|  |  |  | LAQVRK |  | IPVARK | FPLARR |
|  |  |  | LEAARK |  | LPAARK | FPPFRR |
|  |  |  | LEFVRK |  | LPIARK | IPIARK |
|  |  |  | LEIAKK |  | LPIARR | IPIARR |
|  |  |  | LEIARK |  | LPIMRK | IPLARR |
|  |  |  | LEIDRK |  | LPIPRK | IPMQRK |
|  |  |  | LEIVKK |  | LPLARK | IPQARK |
|  |  |  | LEIVRK |  | LPLARR | IPQARR |
|  |  |  | LELVKK |  | LPMARK | LAMARR |
|  |  |  | LPIARK |  | LPMARR | LPFARK |
|  |  |  | LPIGRK |  | LPQARK | LPIARK |
|  |  |  | LPIKRR |  | LPQTRK | LPIARR |
|  |  |  | LPISRK |  | LPVARK | LPIMRR |
|  |  |  | LPITRK |  | LPVARR | LPLARK |
|  |  |  | LPLQRK |  | LPVVRK | LPLARR |
|  |  |  | LPMARK |  | LQVARK | LPLTRR |
|  |  |  | LPQARK |  | LSIARK | LPMARK |
|  |  |  | LQISRR |  | MPIARK | LPMARR |
|  |  |  | LQITKK |  | MPIARR | LPMTRK |
|  |  |  | LQIVRK |  | MPIMRK | LPIMRK |
|  |  |  | LQMTRK |  | MPIPRR | LPIPRR |
|  |  |  | LRITRK |  | MPLARK | LPITRK |
|  |  |  | MEIAKK |  | MPLYRK | LPITRR |
|  |  |  | MEIVKK |  | MPMARK | LPQARK |
|  |  |  | MEIVRK |  | MPVARK | LPQTRK |
|  |  |  | MPVARK |  | PPIARK | LRIARR |
|  |  |  | MSIARK |  | TPVARK | LRQTRK |
|  |  |  | SIARKK |  | VHQSRK | MPIARK |
|  |  |  | VHIGRK |  | VPLARK | MPIARR |
|  |  |  | VPLARK |  | VPQARK | MPIGRK |
|  |  |  | VPQARK |  | VPQARR | MPIKRK |
|  |  |  |  |  | VPVARK | MRYPRR |
|  |  |  |  |  | VRQSRK | MVIARK |
|  |  |  |  |  |  | PKIARK |
|  |  |  |  |  |  | PPIARK |
|  |  |  |  |  |  | PTIARK |
|  |  |  |  |  |  | PTQARR |
|  |  |  |  |  |  | QPIARK |
|  |  |  |  |  |  | VPHARR |
|  |  |  |  |  |  | VPIARR |
|  |  |  |  |  |  | VPQARK |
|  |  |  |  |  |  | VPQARR |
|  |  |  |  |  |  | VPQFRK |
|  |  |  |  |  |  | VPQFRR |
|  |  |  |  |  |  | VPQSRR |
